# THE EYE OF THE STORM: END-OF-LIFE CARE IN NEW YORK CITY DURING THE COVID-19 PANDEMIC: LESSONS FOR THE FUTURE OF HOSPICE

**DOI:** 10.1093/geroni/igad104.0623

**Published:** 2023-12-21

**Authors:** Daniel David, Emily Franzosa, Abraham Brody

## Abstract

Public health containment and mitigation policies in response to the COVID-19 pandemic, including social distancing, infection control, and self-quarantine led to a substantial reduction in transmission of COVID-19. Yet they also brought about significant disruptions in healthcare, particularly care at the end of life. These challenges were amplified in older adults of color and those who contend with differing social determinants of health that impact care outcomes. Hospices played a key role in supporting patients at home throughout the pandemic and yet concomitant with the surge in demand for hospice, there was a gap in hospice delivery, exposing latent vulnerabilities in the capacity to respond to a crisis that demanded a coordinated, synchronized approach. We present the findings of an innovative, mixed methods study funded by the National Institute of Nursing Research that combines qualitative content analysis of linked electronic health records with qualitative stakeholder interviews to gain an in-depth understanding of the challenges of delivering hospice care in NYC during the pandemic. Interviews include the perspectives of nurses, managers, administrators, physicians, medical directors, social workers, and clergy from several healthcare practices. Our presenters address 4 key areas: a) Provider experiences and organizational challenges and successes, b) Challenges in transitioning from primary care to hospice, c) Efforts to provide equity-focused hospice care, and d) The central role of home health aides in end-of-life care. Together, these experiences provide insights into lessons learned and point to new opportunities to design and deliver high-quality hospice care in the future.

